# Supplementary material for: The impact of elevation and population density on dengue incidence and force of infection across the Philippines: Implications for climate-adapted surveillance
Source: PLoS Negl Trop Dis. 2026 May 26;20(5):e0014356. doi: 10.1371/journal.pntd.0014356 (PMC13229340; doi:10.1371/journal.pntd.0014356)
Supplement: S1 Table — (DOCX) [file pntd.0014356.s001.docx]

**S1 Table**: The total population and number of collated and surveyed dengue case reports across the Philippines during the study period.
