# Supplementary material for: The impact of elevation and population density on dengue incidence and force of infection across the Philippines: Implications for climate-adapted surveillance
Source: PLoS Negl Trop Dis. 2026 May 26;20(5):e0014356. doi: 10.1371/journal.pntd.0014356 (PMC13229340; doi:10.1371/journal.pntd.0014356)
Supplement: S1 Fig — (DOCX) [file pntd.0014356.s002.docx]

**
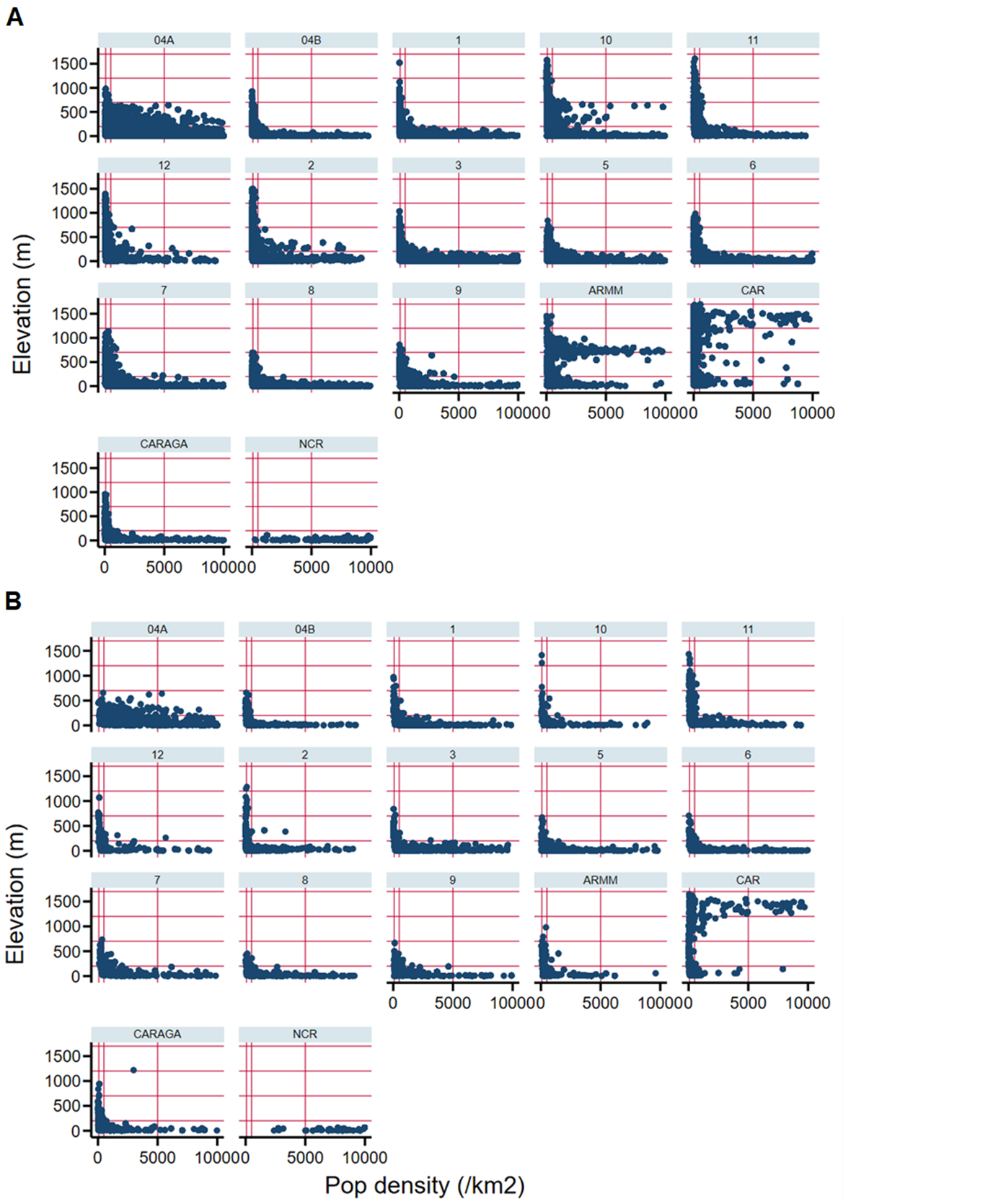
**

**S1 Fig**: The average population densities (km^2^) and elevations (metres) of the collated (**A**) and serologically-surveyed (**B**) dengue case reports between 2013-2018 and 2014-2019 by Philippine regions, respectively. Red lines correspond to geographical strata thresholds.
