# Supplementary material for: The impact of elevation and population density on dengue incidence and force of infection across the Philippines: Implications for climate-adapted surveillance
Source: PLoS Negl Trop Dis. 2026 May 26;20(5):e0014356. doi: 10.1371/journal.pntd.0014356 (PMC13229340; doi:10.1371/journal.pntd.0014356)
Supplement: S2 Fig — (DOCX) [file pntd.0014356.s003.docx]

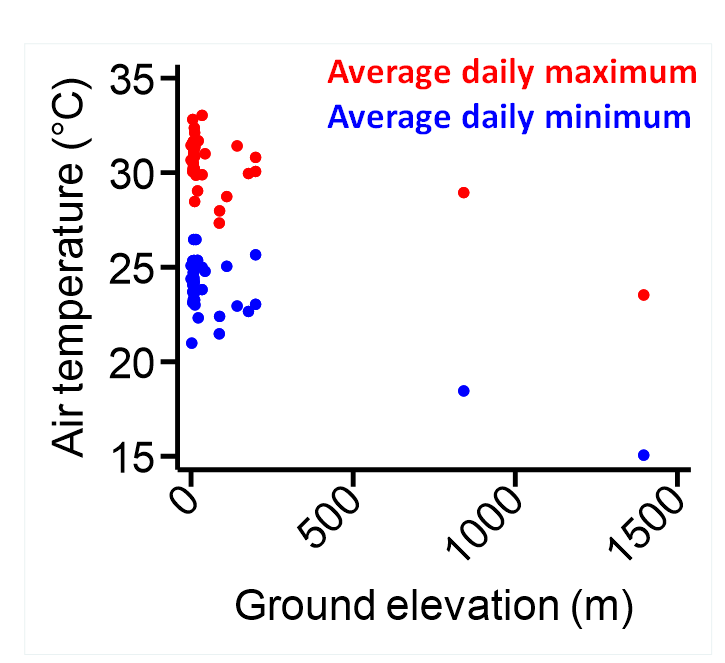


**S2 Fig**: The association between weather station ground elevation (metres) and averaged daily maximum/minimum air temperature between 2010-15. Data obtained from 55 weather stations situated across the Philippines (PAGASA: (PAGASA: Philippine Atmospheric, Geophysical and Astronomical Services Administration; Manila; Philippines).
