# Supplementary material for: The impact of elevation and population density on dengue incidence and force of infection across the Philippines: Implications for climate-adapted surveillance
Source: PLoS Negl Trop Dis. 2026 May 26;20(5):e0014356. doi: 10.1371/journal.pntd.0014356 (PMC13229340; doi:10.1371/journal.pntd.0014356)
Supplement: S3 Fig — (DOCX) [file pntd.0014356.s004.docx]

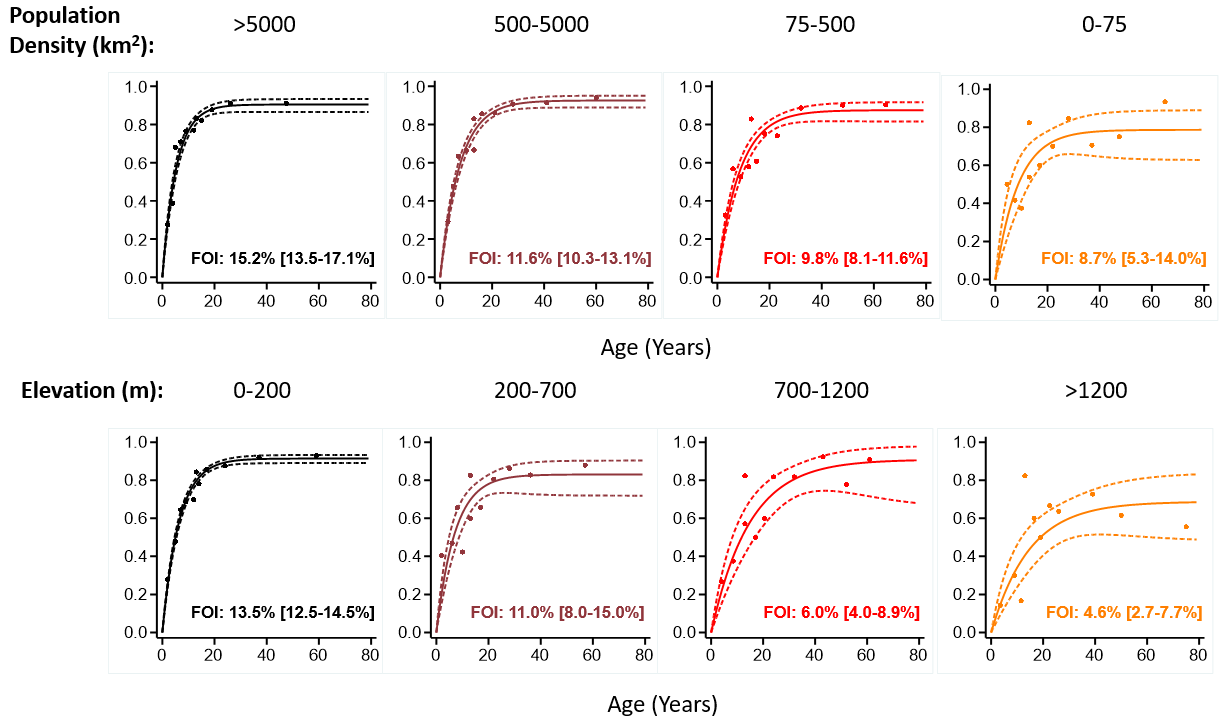


**S3 Fig**: Dengue age-seroprevalence and FOI by elevation and population density strata according to catalytic models. Dots: observed age-seroprevalence, lines: fitted age seroprevalence, dash line: fitted age-seroprevalence 95%CIs.
