# Supplementary material for: The impact of elevation and population density on dengue incidence and force of infection across the Philippines: Implications for climate-adapted surveillance
Source: PLoS Negl Trop Dis. 2026 May 26;20(5):e0014356. doi: 10.1371/journal.pntd.0014356 (PMC13229340; doi:10.1371/journal.pntd.0014356)
Supplement: Attachment — Submitted filename: response to reviewers.docx [file pntd.0014356.s006.docx]

**Response to reviewers**

Dear PLOS NTD editors & Reviewers,

Re:  “The impact of altitude and population density on dengue incidence and force of infection across the Philippines: Implications for climate-adapted surveillance"

PNTD-D-26-00215

We are resubmitting a revised version of our manuscript titled " The impact of altitude and population density on dengue incidence and force of infection across the Philippines: Implications for climate-adapted surveillance " for publication in PLOS NTD. We thank the editors and reviewers for their useful insights and suggestions. We have responded to specific comments below and refer to changes in the revised manuscript. We hope this clarifies any issues raised and look forward to hearing from you.

Best wishes

Joseph Biggs (corresponding author)

**Editor**

**Journal Requirements:**

If the reviewer comments include a recommendation to cite specific previously published works, please review and evaluate these publications to determine whether they are relevant and should be cited. There is no requirement to cite these works unless the editor has indicated otherwise.

**Response:** We thank the editor for this clarification.

1) We do not publish any copyright or trademark symbols that usually accompany proprietary names, eg ©,  ®, or TM  (e.g. next to drug or reagent names). Therefore please remove all instances of trademark/copyright symbols throughout the text, including:
- ® on page: 8.

**Response**: As suggested, we have removed the trademarks symbols.

2) Please upload all main figures as separate Figure files in .tif or .eps format. For more information about how to convert and format your figure files please see our guidelines: 
[https://journals.plos.org/plosntds/s/figures](https://url.uk.m.mimecastprotect.com/s/IcjCC2WMBTyXrGWfBiXS55PbM?domain=journals.plos.org)

**Response:** All figure files have been uploaded as separate TIFF files

3) We have noticed that you have uploaded Supporting Information files, but you have not included a list of legends. Please add a full list of legends for your Supporting Information files after the references list.

**Response**: As suggested we have now added a full list of legends for the supporting information files after the references list.

4) Some material included in your submission may be copyrighted. According to PLOSu2019s copyright policy, authors who use figures or other material (e.g., graphics, clipart, maps) from another author or copyright holder must demonstrate or obtain permission to publish this material under the Creative Commons Attribution 4.0 International (CC BY 4.0) License used by PLOS journals. Please closely review the details of PLOSu2019s copyright requirements here: PLOS Licenses and Copyright. If you need to request permissions from a copyright holder, you may use PLOS's Copyright Content Permission form.
Please respond directly to this email and provide any known details concerning your material's license terms and permissions required for reuse, even if you have not yet obtained copyright permissions or are unsure of your material's copyright compatibility. Once you have responded and addressed all other outstanding technical requirements, you may resubmit your manuscript within Editorial Manager. 
Potential Copyright Issues:
i) Figure 1. Please (a) provide a direct link to the base layer of the map (i.e., the country or region border shape) and ensure this is also included in the figure legend; and (b) provide a link to the terms of use / license information for the base layer image or shapefile. We cannot publish proprietary or copyrighted maps (e.g. Google Maps, Mapquest) and the terms of use for your map base layer must be compatible with our CC BY 4.0 license.
Note: if you created the map in a software program like R or ArcGIS, please locate and indicate the source of the basemap shapefile onto which data has been plotted.
If your map was obtained from a copyrighted source please amend the figure so that the base map used is from an openly available source. Alternatively, please provide explicit written permission from the copyright holder granting you the right to publish the material under our CC BY 4.0 license.
If you are unsure whether you can use a map or not, please do reach out and we will be able to help you. The following websites are good examples of where you can source open access or public domain maps:
* U.S. Geological Survey (USGS) - All maps are in the public domain. ([http://www.usgs.gov](http://url.uk.m.mimecastprotect.com/s/w4xUC3wNDcNl3MotDsJSQl4Hf?domain=usgs.gov))
* PlaniGlobe - All maps are published under a Creative Commons license so please cite u201cPlaniGlobe, [http://www.planiglobe.com](http://url.uk.m.mimecastprotect.com/s/KhkYC4LO0CkOPWEUVtOS4TM6C?domain=planiglobe.com), CC BY 2.0u201d in the image credit after the caption. ([http://www.planiglobe.com/?lang=enl](http://url.uk.m.mimecastprotect.com/s/0iuMC5LPGCN78K3t8uRSkjyNc?domain=planiglobe.com))
* Natural Earth - All maps are public domain. ([http://www.naturalearthdata.com/about/terms-of-use/](http://url.uk.m.mimecastprotect.com/s/Oxx1C69QJSwvk97UBCqS57rip?domain=naturalearthdata.com)).

**Response**: As requested, we have changed the figure 1 caption to better highlight the source/licence of the shapefile used to generate the map figure. Figure 1 caption reads as follows:

***“Figure 1: Study Location.*** ***A****: Regions of the Philippines. CAR: Cordillera Administrative Region; NCR: National Capital Region; ARMM: Autonomous Region in Muslim Mindanao. B: Major island groups of the Philippines.* ***C****: Average altitude (metres) and population density (individuals/km^2^) per barangay across the Philippines. The country is divided into three main island groups: Luzon, Visayas and Mindanao, further divided into 17 regions, 1,488 municipalities and 42,046 barangays. Base map shapefile (Philippines administrative boundaries) obtained from the Humanitarian Data Exchange (HDX), provided by the United Nations Office for the Coordination of Humanitarian Affairs (OCHA):* [*https://data.humdata.org/dataset/cod-ab-phl*](https://data.humdata.org/dataset/cod-ab-phl)*. Data are available under an open license compatible with CC BY.”*

5) In the online submission form, you indicated that The datasets used in this study are available from the corresponding author on reasonable request.. All PLOS journals now require all data underlying the findings described in their manuscript to be freely available to other researchers, either
1. In a public repository
2. Within the manuscript itself
3. Uploaded as supplementary information.
This policy applies to all data except where public deposition would breach compliance with the protocol approved by your research ethics board. If your data cannot be made publicly available for ethical or legal reasons (e.g., public availability would compromise patient privacy), please explain your reasons by return email and your exemption request will be escalated to the editor for approval. Your exemption request will be handled independently and will not hold up the peer review process, but will need to be resolved should your manuscript be accepted for publication. One of the Editorial team will then be in touch if there are any issues.

**Response**: As suggested above, we previously contacted the PLOS NTD editorial team for further guidance on this issue. We explained that the data included in this manuscript was provided by the department of health epidemiology bureau of the Philippines and The Research institute for Tropical Medicine and represents disease surveillance data that is not publicly available and data sharing requires institutional approval and governance restrictions.

In response to our explanation, the PLOS NTD editor replied *“In this instance it seems there may be acceptable restrictions in place that prevent the public sharing of your minimal data. However, in line with our goal of ensuring long-term data availability to all interested researchers, PLOS’ Data Policy states that authors cannot be the sole named individuals responsible for ensuring data access:*[*https://journals.plos.org/plosntds/s/data-availability#loc-acceptable-data-access-restrictions*](https://journals.plos.org/plosntds/s/data-availability#loc-acceptable-data-access-restrictions)*. Data requests to a non-author institutional point of contact, such as a data access or ethics committee, helps guarantee long term stability and availability of data. Providing interested researchers with a durable point of contact ensures data will be accessible even if an author changes email addresses, institutions, or becomes unavailable to answer requests. Please revise your Data and Code Availability statement accordingly at next resubmission, should your paper be set back to you for revisions.”*

In accordance with the journal, we have updated the data sharing statement to read as follows:

“*The data underlying this study were collected as part of routine surveillance activities and are subject to ethical and governance restrictions, and the conditions under which these data were collected did not include provision for public data sharing. Case report surveillance data are held by the Epidemiology Bureau, Department of Health Philippines, and serological surveillance data are held by the Research Institute for Tropical Medicine (RITM); these data are not publicly available. Researchers who meet the criteria for access to confidential data may submit requests to the respective institutions (Department of Health Philippines: dohosec@doh.gov.ph; Research Institute for Tropical Medicine: phu@ritm.gov.ph), and access is subject to institutional approval and data governance requirements*.”

6) Please provide a detailed Financial Disclosure statement. This is published with the article. It must therefore be completed in full sentences and contain the exact wording you wish to be published.
1) Please clarify all sources of financial support for your study. List the grants, grant numbers, and organizations that funded your study, including funding received from your institution. Please note that suppliers of material support, including research materials, should be recognized in the Acknowledgements section rather than in the Financial Disclosure
2) State the initials, alongside each funding source, of each author to receive each grant. For example: "This work was supported by the National Institutes of Health (####### to AM; ###### to CJ) and the National Science Foundation (###### to AM)."
3) State what role the funders took in the study. If the funders had no role in your study, please state: "The funders had no role in study design, data collection and analysis, decision to publish, or preparation of the manuscript."
4) If any authors received a salary from any of your funders, please state which authors and which funders..
If you did not receive any funding for this study, please simply state: u201cThe authors received no specific funding for this work.u201d

7) Your current Financial Disclosure states, "The author(s) received no specific funding for this work.".
However, your funding information on the submission form indicates receiving funds. 
Please indicate by return email the full and correct funding information for your study and confirm the order in which funding contributions should appear. Please be sure to indicate whether the funders played any role in the study design, data collection and analysis, decision to publish, or preparation of the manuscript.

**Response**: As requested we have updated the financial statement which now reads as follows: *“The authors received no specific funding for this work. J.C.R.H. was supported by the The Royal Society (grant CHG\R1\170061). M.L.H. and C.D.P. were supported by the Newton Fund Institutional Links programme (project number 216416089), delivered by the British Council and the Commission on Higher Education. The funders had no role in study design, data collection and analysis, decision to publish, or preparation of the manuscript.”*

**Reviewers' comments:**

Reviewer's Responses to Questions

**Key Review Criteria Required for Acceptance?**
As you describe the new analyses required for acceptance, please consider the following:
**Methods**
-Are the objectives of the study clearly articulated with a clear testable hypothesis stated?

-Is the study design appropriate to address the stated objectives?

-Is the population clearly described and appropriate for the hypothesis being tested?

-Is the sample size sufficient to ensure adequate power to address the hypothesis being tested?

-Were correct statistical analysis used to support conclusions?

-Are there concerns about ethical or regulatory requirements being met?

Reviewer #1: -Are the objectives of the study clearly articulated with a clear testable hypothesis stated? YES
-Is the study design appropriate to address the stated objectives? YES
-Is the population clearly described and appropriate for the hypothesis being tested? YES
-Is the sample size sufficient to ensure adequate power to address the hypothesis being tested? YES
-Were correct statistical analysis used to support conclusions? Beyond my expertise.
-Are there concerns about ethical or regulatory requirements being met? NO.

Reviewer #2: The objectives of the study are clear, the study has a very robust sample size, the population is well described and appropriate, and the statistics are rigorous. However, I do have the following suggestions/concerns:

With regard to serology, the authors note that sampled individuals have similar demographic characteristics to dengue cases, but do they have similar geographic distribution?

**Response**: We agree that the distribution of the reported and surveyed dengue case reports is an important consideration that needs clearly addressing in this article. In S1 figure, we clearly demonstrate that both collated and surveyed case reports are from similar regions (and similar geographical strata within each region). We have updated the results section to better clarify this: *“The collated and surveyed dengue case reports included in this study exhibited similar distributions, as they were derived from comparable geographic strata across similar regions (S1 Fig).”* **(see section results: lines X-X)**

Figure 1: Panel B is not legible and quite possibly not necessary.

**Response**: Panel B of figure one shows all the geographical regions of the Philippines. WE have updated figure 1 to make this panel clearer and legible (see figure 1). We believe including a panel showing the regions of the Philippines is necessary as later in the paper we refer to specific results from given regions.

Fig S2 does not instill much confidence in the association between elevation and temperature, given the scant number of temperature measurements above 500 m. Although temperature is certainly known to decrease with increasing elevation, is there an alternative source of more densely sampled data to better reveal this association in the Philippines? I do not think the figure is a deal breaker for the study, but its limitations should certainly be discussed if alternative data is not available.

**Response**: We agree, the clear association between temperature and elevation – and the evidence to support this association – needs to clarified In this study. We now cite a study conducted in the Philippines that clearly demonstrates the relationship. The methods section now reads as follows: “Geographical data were obtained at the local barangay level. A 2015, 100-metre resolution Digital Altitude Model (USGS Earth Explorer; USA) was used to estimate the average barangay altitude (metres) in ArcGIS (v10.5). In the Philippines, the association between decreasing temperature and increasing elevation has been well documented [20].”

[20] Olaguera, L. M. P., E. K. Badua, A. G. A. Llorin, F. A. T. Cruz, J. R. T. Villarin, J. A. Manalo, M. Q. Villafuerte II, and J. Matsumoto, 2025: Rainfall and temperature-based Philippine climate zones using Self-Organizing Maps. SOLA, 21C, 10−20, doi:10.2151/sola.21C-002.

**Results**


-Does the analysis presented match the analysis plan?

-Are the results clearly and completely presented?

-Are the figures (Tables, Images) of sufficient quality for clarity?

Reviewer #1: -Does the analysis presented match the analysis plan? YES
-Are the results clearly and completely presented? YES
-Are the figures (Tables, Images) of sufficient quality for clarity? YES

Reviewer #2: The analysis is robust, the results are clearly presented (though see some suggestions in minor critiques) and the figures are clear.

**Conclusions**


-Are the conclusions supported by the data presented?

-Are the limitations of analysis clearly described?

-Do the authors discuss how these data can be helpful to advance our understanding of the topic under study?

-Is public health relevance addressed?

Reviewer #1: -Are the conclusions supported by the data presented? YES
-Are the limitations of analysis clearly described? YES
-Do the authors discuss how these data can be helpful to advance our understanding of the topic under study? YES
-Is public health relevance addressed? YES

Reviewer #2: Generally the conclusions are well supported, the limitations are discussed, and the public health relevance is well articulated. I do, however, suggest the following changes:

1. Overall
It is not necessary to frame population density as a proxy for urbanization- population density is population density and important in its own right. Furthermore, population density may sometime be decoupled from other important markers of urbanization, such as NDBI. And lastly, the authors toggle back and forth between referring to population density and urbanization, even in the abstract, which is quite confusing. I recommend that urbanization be struck throughout the manuscript and replaced with population density.

**Response**: Upon reflection, we fully agree with this reviewers recommendation and have replaced the term urbanisation with population density as these should not be considered interchangeable metrics. See changes throughout the paper.

Moreover, We have replaced the term ‘altitude’ with ‘elevation’ throughout the manuscript to accurately reflect that we are referring to the height of geographic locations above sea level, rather than vertical measurements above the ground or atmosphere.

2. Abstract
Delete second sentence; it is neither necessary nor entirely correct

**Response**: We have adapted the second sentence to better clarify the importance of this study. It now reads as follows: “Climate change is accelerating the geographical expansion of Aedes mosquito vectors, facilitating the emergence of arboviral diseases such as dengue in new regions. However, there is limited understanding of how environmental factors, such as temperature and population density, differentially influence key metrics of dengue transmission.” (**See abstract**)

**Editorial and Data Presentation Modifications?**


Use this section for editorial suggestions as well as relatively minor modifications of existing data that would enhance clarity. If the only modifications needed are minor and/or editorial, you may wish to recommend “Minor Revision” or “Accept”.

Reviewer #1: Please see summary and general comments.

Reviewer #2: Abstract
Change first sentence to
“Climate change is enabling geographic expansion of Aedes mosquito vectors into new areas.

**Response**: As suggested we have included the term geographical expansion and updated the first sentence of the abstract to read: *“Climate change is accelerating the geographical expansion of Aedes mosquito vectors, facilitating the emergence of arboviral diseases such as dengue in new regions.”*

Line 78: Add “than incidence” after “reliable metric”

**Response**: This sentence has been removed to accommodate the changes explained above.

Introduction
Rephrase the second sentence for correctness- not all Aedes species serve as dengue vectors, nor are all Aedes species found in warm, humid climates. Furthermore, even among the major dengue vectors, Ae. albopictus is more common at urban edges than in urban habitats proper.

Suggest you consider this paper, which shows higher Ae. aegypti abundance in rural than urban land covers:
Higher abundance of the vector Aedes aegypti in rural areas than in urban areas in Managua, Nicaragua.
Laguna HS, Díaz JM, Lopez MM, Balmaseda A, Harris E, Coloma J, Juarez JG.Res Sq [Preprint]. 2025 Mar 12:rs.3.rs-6059011. doi: 10.21203/rs.3.rs-6059011/v1.

**Response**: We agree with the introductory sentence was overgeneralised. We have changed it to “Dengue virus (DENV) is primarily transmitted by *Aedes* mosquitoes, particularly *Ae. aegypti* and *Ae. albopictus*, which thrive in warm, humid environments and are well adapted to urban settings.”

Results

Figure 2F- need to specify that age is in years

**Response**: Agreed and have made this change to figure 2F

Paragraph starting on line 284: First sentence of the paragraph contradicts the last sentence of the paragraph.

**Response**: we agree the original first and last sentence of this paragraph was contradictory. We now mention there is a modest relationship instead of an association: The whole paragraph now reads: “In contrast, a modest relationship was observed between population density and dengue incidence. Incidence increased slightly with population density, from 1.06 cases/1,000/year in barangays averaging <75 individuals/km^2^ to 1.88 cases/1,000/year in barangays averaging >5,000 individuals/km^2^ (Fig. 2A). This trend persisted when stratified by island group and year, although exceptions were noted in Luzon and certain years (Fig. 2B & C). Overall, these findings suggest that dengue incidence trends are spatially and temporally sporadic across the Philippines, with no consistent association with either elevation or population density.”

Figure 2D – reversing the orientation of elevation (high to low from left to right) relative to the panel above it (low to high from left to right) is problematic and will lead to misinterpretation- strongly suggest the x-axis be flipped to read from low to high.

**Response**: We agree with this suggestion and have reversed the x-axis on Figure 2D to prevent misinterpretation.

Discussion
Line 387: Add FOI at the start of the sentence

**Response**: Upon reviewing the sentence structure, we believe the current structure is the clearest: *“In this nationally-representative study across the Philippines, we examined the independent and synergistic effects of elevation and population density on reported dengue incidence and DENV FOI at fine spatial scales.”*

Line 398: The sentence that starts on this line is a run-on; also “later” needs to be amended to “latter”.

**Response:** As suggested we have revised this paragraph for clarity. It now reads as *“Results highlight that despite being highly heterogenous at fine spatial scales, overall dengue transmission intensity is highest in low-lying urban centres, and population density is less of a driver of dengue transmission in high-elevation areas. The latter is likely a consequence of cooler temperatures being a limiting factor of Aedes mosquitoes being able to proliferate and spread dengue effectively, irrespective of the density of human hosts in the vector’s vicinity”*

**Summary and General Comments**


Use this section to provide overall comments, discuss strengths/weaknesses of the study, novelty, significance, general execution and scholarship. You may also include additional comments for the author, including concerns about dual publication, research ethics, or publication ethics. If requesting major revision, please articulate the new experiments that are needed.

Reviewer #1: The authors compare the impact of altitude and population density on dengue incidence and force of infection across the Philippines, concluding that FOI is a more reliable metric for assessing transmission intensity than approaches that depend upon accurate clinical case reporting.

The manuscript is clearly presented but would benefit from additional references. I have highlighted some examples below, but I encourage the authors to review the text to ensure that key statements are appropriately supported.

**Response**: We have reviewed the text as suggested and have added supporting references when appropriate. For instance we now provide a reference that clearly demonstrates the relationship between elevation and temperature across the Philippines.

The authors use the terms ‘altitude’ and ‘elevation’ interchangeably in the text, tables, and figures throughout the manuscript and supplementary materials. They may wish to settle on one of these terms. McVicar et al. provide a nice concise definition of each in the following manuscript: DOI 10.1007/s00442-012-2416-7

**Response**: We agree with this statement and have settled on the term ‘elevation’ throughout the paper for clarity. We have replaced the term ‘altitude’ with ‘elevation’ throughout the manuscript to accurately reflect that we are referring to the height of geographic locations above sea level, rather than vertical measurements above the ground or atmosphere.

If permitted, for reasons of transparency and ease of access, I encourage the authors to share their data either as supplementary material or via a data repository. Otherwise, I only have a few small comments and suggestions for the authors.

**Response**: We thank the reviewer for this suggestion and have clarified the reasons for not sharing the data. The data sharing statement now better explains this: “*The data underlying this study were collected as part of routine surveillance activities and are subject to ethical and governance restrictions, and the conditions under which these data were collected did not include provision for public data sharing. Case report surveillance data are held by the Epidemiology Bureau, Department of Health Philippines, and serological surveillance data are held by the Research Institute for Tropical Medicine (RITM); these data are not publicly available. Researchers who meet the criteria for access to confidential data may submit requests to the respective institutions (Department of Health Philippines: dohosec@doh.gov.ph; Research Institute for Tropical Medicine: phu@ritm.gov.ph), and access is subject to institutional approval and data governance requirements*.”

Short title
Line 4: I suggest using key terms that agree with the title i.e., ‘population density’ rather than ‘urbanisation’, which has a slightly different meaning (something that is mentioned in the opening sentences of the abstract).

**Response**: We fully agree with this suggestion and have removed terms urbanisation and instead replaced them with population density as they represent different metrics.

Abstract
Lines 78 – 79: For clarity, I suggest changing the text to “FOI provides a more reliable metric than incidence for understanding transmission dynamics and guiding interventions”.

**Response**: We agree and have changed the abstract to include: “FOI is a more reliable metric than incidence for understanding transmission dynamics and guiding interventions.”

Introduction
Line 122: Please include an appropriate reference at the end of the first sentence.

**Response**: This now sentence has a relevant reference: “Climate change is reshaping global health risks by accelerating the spread of vector-borne diseases such as dengue [1]”

Messina, J.P., Brady, O.J., Golding, N. *et al.* The current and future global distribution and population at risk of dengue. *Nat Microbiol* **4**, 1508–1515 (2019). https://doi.org/10.1038/s41564-019-0476-8

Lines 123 – 124: Not all Aedes species mosquitoes transmit dengue, so perhaps change to “Certain Aedes species mosquitoes, which transmit the dengue virus…” and include a suitable reference. In a word or two, you could also be clearer about the location of the warm, humid, urban environments to which you are referring e.g., the tropics, subtropics, or elsewhere.

**Response**: We agree and have updated the sentence to now read as*: “Dengue virus (DENV) is primarily transmitted by Aedes mosquitoes, particularly Ae. aegypti and Ae. albopictus, which thrive in warm, humid environments and are well adapted to urban settings”*

Lines 133 – 134: As it is written, it seems like population densities are discrete units that need to be in proximity for transmission to occur. It may be more accurate to state 'High human population densities facilitate sustained dengue transmission between vectors and human hosts'.

**Response**: Agreed and have changed the sentence to read exactly as suggested.

Line 137 - 139: Please change “vectorial competence” to “vector competence”, which is the more commonly used term, and include a citation at the end of this sentence (that is assuming that you are referring to vector competence and not vectorial capacity – please do not confuse these terms, which might be worth briefly defining). Please also clarify the “daily fluctuations” to which you are referring in the final part of the sentence e.g., average/mean temperature, temperature range, or something else.

**Response**: Agree this sentence needs to be clearer: It now reads as: “Urban environments with abundant standing water in artificial containers, gutters, and storm drains, provide ideal breeding sites for *Aedes* mosquitoes [7, 8]. Air temperature also plays a critical role in influencing their abundance, survival, and vectorial capacity. The optimal temperature for maximum vectorial capacity is estimated at around 29 °C, though this may vary with daily fluctuations in temperature [9].

Methods
Line 188 – Please include a reference to the WHO 2008 criteria at the end of this sentence. I think it would also be beneficial if you briefly explained the meaning of ‘dengue warning signs’, since the term is used throughout the manuscript.

**Response**. We agree with this suggestion and included an appropriate reference and included the meaning of dengue with warning signs: It now reads as follows: “Cases are classified according to the WHO criteria as dengue without warning signs, dengue with warning signs, or severe dengue. Dengue with warning signs is a symptomatic dengue infection accompanied by clinical indicators, such as abdominal pain, persistent vomiting, fluid accumulation, mucosal bleeding, lethargy, and/or liver enlargement [21].”

[20] World Health Organization, 2009. *Dengue: Guidelines for diagnosis, treatment, prevention and control*. 2nd ed. Geneva: World Health Organization. Available at: <https://iris.who.int/handle/10665/44188>

Results
Lines 261 – 263: The opening sentence of the results is slightly convoluted and could be refined. You mentioned the years of collection in the methods so you might consider excluding this information, and it is clear at this stage that the study is being conducted in the Philippines, so it doesn’t need to be mentioned twice in this sentence.

**Response**: Agreed, we have changed the sentence to read as: “A total of 1,112,317 dengue case reports from 2013–2018, along with 22,270 serologically surveyed case reports from 2014–2019, covering the entire Philippines, were included in this study.”

Lines 272 – 273: Is this statistically significant? It appears that only two of the weather stations were above about 250 m elevation in Figure S2.

**Response**: The reviewer highlights this sentence: “Lastly, lower daily maximum and minimum air temperatures (2010-2015 data) were observed in areas with higher altitudes (S2 Fig).” We refrained from conducting any statistical tests on these data as the association between elevation and temperature has been well characterised globally, and in the Philippines. We now additionally reference a specific scientific article in the methods that highlights this association with more robust analysis. Thus we believe it not appropriate to conduct statistical tests to similarly show this association.

Line 338 – 339: Since this subheading is “The combined impact…” you might wish to change the previous subheading to “The individual impact…” (or something appropriate) to more clearly define the separate analyses.

**Response**: We agree with this suggestion. The results subheading now reads as:” The individual impact of elevation and population density on dengue incidence and force of infection”

Discussion
Line 391: You may wish to amend the text slightly to highlight that ‘resources’ are often limited for combatting VBDs e.g., “to allocate limited resources to control interventions”.

**Response**: We agreed and have amended this sentence to now read as: “This key mismatch illustrates the challenges of characterising dengue transmission dynamics based on case surveillance data alone and reinforces the value of utilising alternative metrics of dengue transmission intensity to allocate limited control intervention resources, monitor changes in transmission and identify areas at risk of future outbreaks.”

Line 399: Please refer specifically to relevant vectors (e.g., Ae. aegypti/albopictus) here, since other Aedes species are more tolerant of cooler temperatures, though they aren’t necessarily vectors. Also change “later” to “latter”.

**Response**: We agree with this suggestion and have changed the sentence to read as: Results highlight that despite being highly heterogenous at fine spatial scales, overall dengue transmission intensity is highest in low-lying urban centres, and population density is less of a driver of dengue transmission in high-elevation areas. The latter likely reflects cooler temperatures limiting the ability of *Ae. aegypti* and *Ae. albopictus* to proliferate and efficiently transmit dengue, regardless of local human population density [9, 24].

Figures
Fig 1. You use elevation/altitude interchangeably in the map and caption. Please be consistent in use of terminology.

**Response:** As previously suggested the word altitude in this paper has been replaced with elevation.

Fig 1. The numbers and abbreviations in panel B are not defined in the caption (although some are later mentioned in the text). I suggest also defining them in the figure caption.

**Response:** Figure 1B caption has been updated to reflect these numbers are regions and spells out the abbreviations. It now reads as: ..”Regions of the Philippines. CAR: Cordillera Administrative Region; NCR: National Capital Region; ARMM: Autonomous Region in Muslim Mindanao”

Fig 2. I noticed you flipped the x-axis for ‘Elevation’ in Panel D, and I understand that you have done this to show a left to right increase in exposure to DENV IgG. However, it is easy to miss. I suggest either maintaining the same direction (low to high elevation) shown in the other panels for elevation OR mentioning the change in direction/order in the figure caption.

**Response**. We agree with this suggestion and have correctly ordered the x axis of Fig2D (See figure 2)

Fig 3. A small legend for the colors in panels A and B would be nice. There is space for this, and it would help with quick interpretation of the figure.

**Response**: We have included a colour ramp legend to Figure 3 as suggested for clarity (see Figure 3)

Reviewer #2: This study interrogates the association of DENV transmission with both population density and elevation (a proxy for temperature) in the Philippines. The results reveal that both factors influence force of infection, but that these effects are not apparent when analyzing passively reported DENV cases. Furthermore, the study finds that the average age of dengue with warning signs cases correlates well with FOI, offering a more economical measure to track DENV transmission. The study is valuable in its country-wide scope, thorough analysis of FOI and incidence, and findings with translational relevance for dengue monitoring.
